# Isolated metastases of hepatocellular carcinoma in the right atrium: Case report and review of the literature

**DOI:** 10.3892/ol.2013.1240

**Published:** 2013-03-08

**Authors:** MANRI KAWAKAMI, MASAHIKO KODA, MARI MANDAI, KEIKO HOSHO, YOSHIKAZU MURAWAKI, WAKAKO ODA, KAZUHIKO HAYASHI

**Affiliations:** 1 Department of Multidisciplinary Internal Medicine, Division of Medicine and Clinical Science, School of Medicine, Yonago 683-8504, Japan;; 2 Division of Molecular Pathology, Tottori University, Yonago 683-8504, Japan

**Keywords:** hepatocellular carcinoma, isolated atrial metastasis, prognosis

## Abstract

The aim of this study was to clarify the clinical features of patients with isolated HCC metastases to the heart. A 66-year-old female hospitalized with a hepatocellular carcinoma (HCC) ranging from the right to the left lobe and with a tumor thrombus in the main portal vein, was treated with intraarterial cisplatin, 5-fluouracil, adriamycin and mitomycin. Computed tomography (CT) one month later revealed that the HCC had progressed with multiple lung metastases and moderate ascites. The patient had no symptoms. Magnetic resonance imaging (MRI) and echocardiography revealed a round, movable tumor with a diameter of 2 cm in the right atrium (RA). The patient succumbed to HCC five months later. An autopsy revealed HCC with portal tumor thrombi and metastases to the lungs, inferior vena cava (IVC) and RA. The metastases in the RA and IVC were not continous with the intrahepatic tumor and were histologically attached to the endocardium and endothelium, respectively. An isolated metastasis of a HCC of the RA and IVC is extremely rare. In conclusion, although the majority of isolated metastases of HCC to the heart were diagnosed by echocardiography and were treated with mainly surgery, they had poor prognosis. The echocardiography should be performed for patients with advanced HCC. A novel treatment including molecular targeted drugs is required.

## Introduction

Hepatocellular carcinomas (HCCs) frequently invade the vascular system at points such as the portal and hepatic veins. The results of autopsy studies indicate a 2.7–4.1% incidence of atrial metastases of HCC (1,2). A correct diagnosis is important in the clinical setting since cardiac metastases are able to induce sudden cardiac arrest. The majority of metastases develop as continuous extensions of a tumor thrombus in the hepatic vein. However, isolated cardiac metastases are extremely rare. The present study describes a 66-year-old female with an isolated right atrial metastasis of a HCC and reviews previous published studies, treatments and outcomes in similar patients. Written informed consent was obtained from the patients’ family.

## Case report

A 66-year-old female was diagnosed with chronic hepatitis type B and HCC in May 2004. The patient had no jaundice, vascular spiders, palmar erythema or cardiac murmurs. A hard mass was palpable from the right hypochondrium to the epigastrium. The laboratory results (normal ranges in parentheses) were as follows: total bilirubin, 0.9 mg/dl (0.2–1.2 mg/dl); aspartate aminotransferase, 115 IU/l (5–40 IU/l); alanine aminotransferase, 105 IU/l (5–47 IU/l); alkaline phosphatase 491 IU/l (111–295 IU/l); and lactate dehydrogenase, 199 IU/l (100–225 IU/l). The patient was positive for the hepatitis B surface antigen and e-antibodies. The serum α-fetoprotein and des-γ-carboxy prothrombin levels were elevated to 687,460 ng/ml (normal range, <13.2 ng/ml) and 1037 mAU/ml (normal range, <40 mAU/ml), respectively.

**Figure 1:**
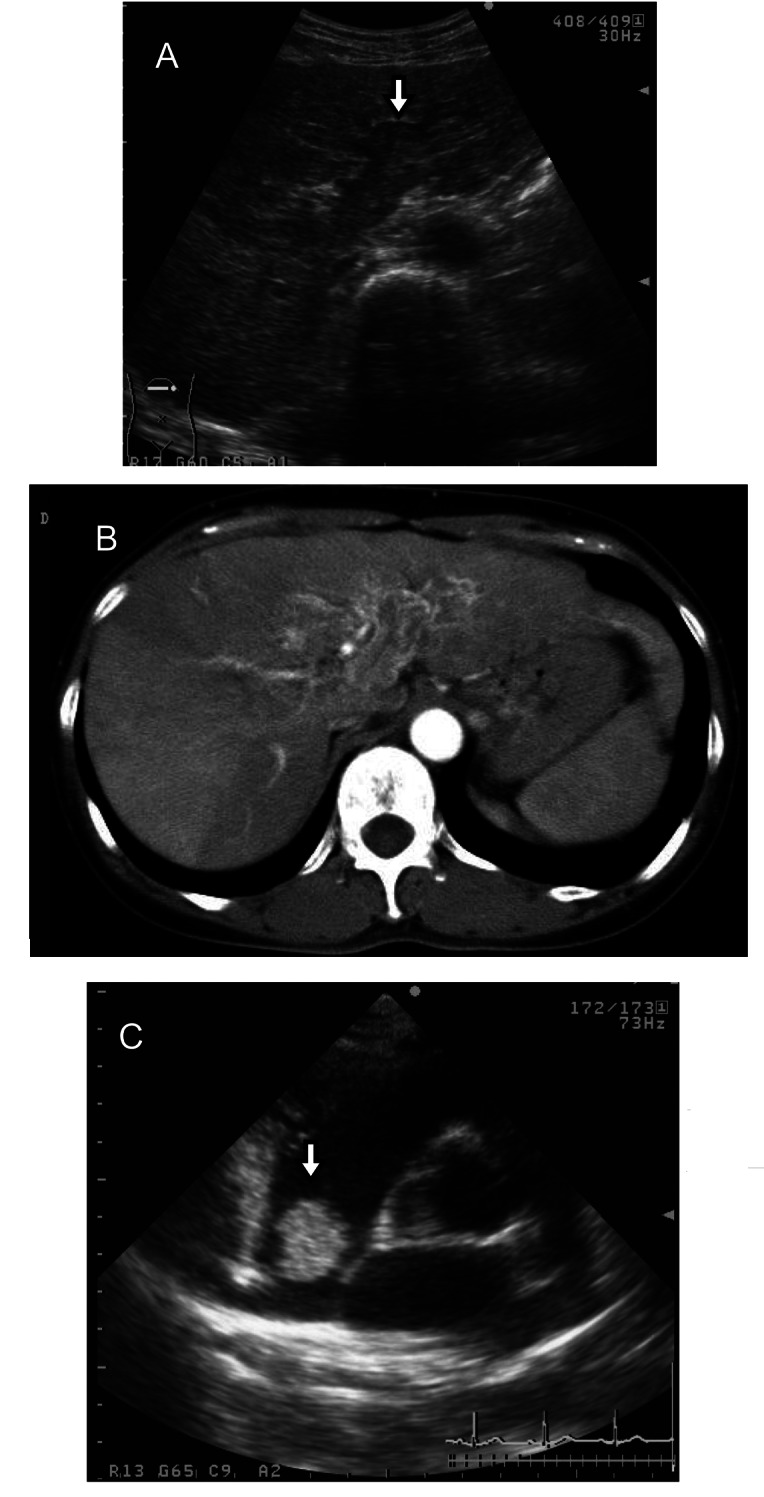
Imaging findings. (A) Abdominal sonographic image showing ill-defined large isoechoic mass with portal thrombus (arrow). (B) Computed tomography (CT) image showing ill-defined large heterogeneous mass occupying the bilateral liver lobes, and portal thrombus accompanying a cavernous transformation. (C) Transthoracic echocardiography image showing a round, movable tumor of a 2-cm diameter in the right atrium (RA; arrow).

Abdominal sonography and computed tomography (CT) imaging revealed a large mass reaching from the right to the left lobe and a tumor thrombus in the main portal vein (Fig. 1A and B). Angiography revealed a hypervascular tumor in the right lobe exhibiting the thread and streak sign. No metastases were identified in the right atrium (RA) or inferior vena cava (IVC) prior to starting intraarterial chemotherapy with cisplatin, 5-fluouracil, adriamycin and mitomycin.

**Figure 2:**
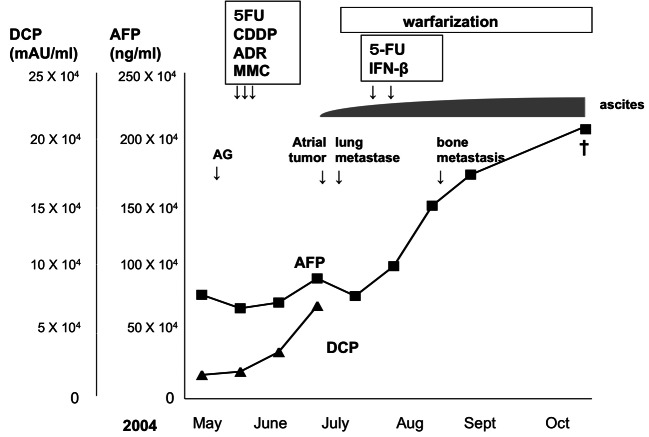
Clinical course of the patient. ADR, adriamycin; AFP, α-fetoprotein; AG, angiography; CDDP, cisplatin; DCP, des-γ-carboxy prothrombin; 5-FU, 5-fluouracil; IFN-β, interferon β; MMC, mitomycin C.

An enhanced CT in July, 2004, showed that the HCC had progressed and that multiple lung metastases had developed with moderate ascites. Magnetic resonance imaging (MRI) and echocardiography revealed a round, movable tumor with a diameter of 2 cm in the RA (Fig. 1C), but no tumor thrombus in the IVC. The atrial tumor was not continuous with the intrahepatic HCC. Anticoagulation therapy with warfarin was administered, however the patient succumbed to hepatic failure five months later (Fig. 2).

**Figure 3:**
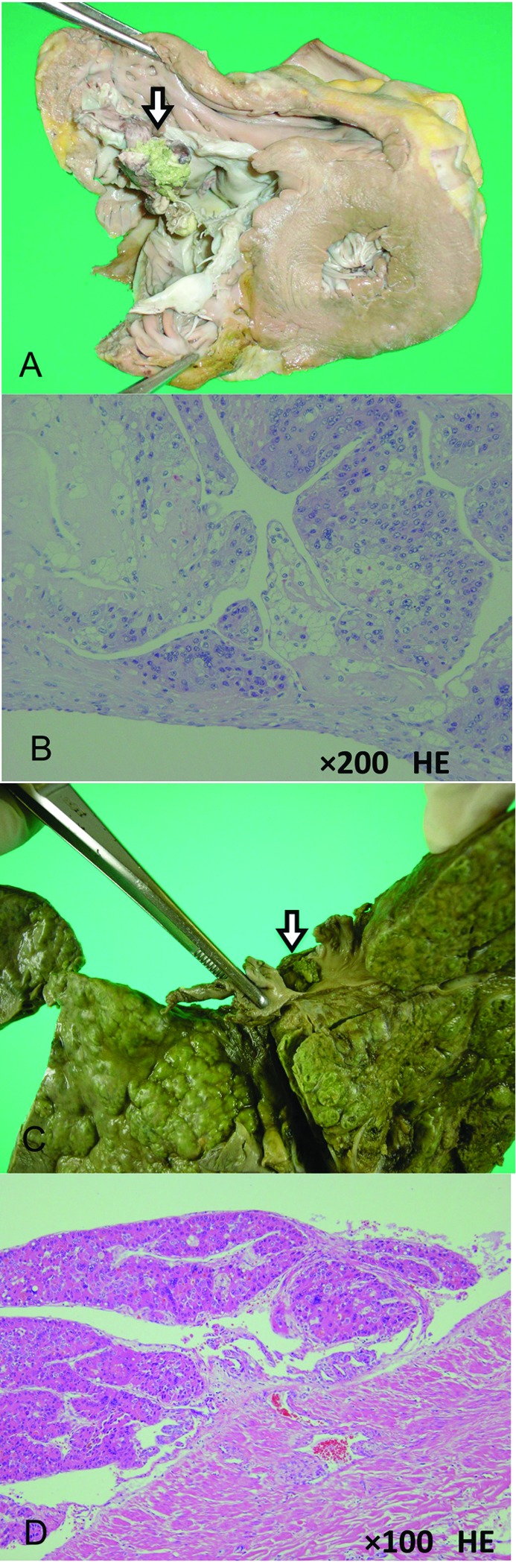
Appearance and diagnosis of autopsy specimen. (A) Yellowish tumor with irregular surface (arrow) located in the right atrium (RA) of the heart specimen at autopsy. (B) Right atrial tumor attached to the endocardium diagnosed as a moderately differentiated HCC of trabecular type (hematoxylin-eosin stain). (C) Greenish tumor with irregular surface (arrow) located in inferior vena cava (IVC), discontinuous with the intrahepatic HCC. (D) Tumor attached to the endothelium in the IVC, pathologically diagnosed as a moderately differentiated HCC of trabecular type (hematoxylin-eosin stain). HCC, hepatocellular carcinoma.

An autopsy revealed diffuse-type HCC in the bilateral lobes of the liver that weighed 1,365 g, with a tumor thrombus in the main trunk of the portal vein, bilateral multiple lung metastases and tumor thrombi in the artery of the right upper lung. A yellowish irregular-surfaced mass with a diameter of 10 mm located in the RA, and a similar small independent mass in the IVC (Fig. 3), were discontinuous with the intrahepatic HCC. Histologically, the intrahepatic and right atrial tumors were moderately differentiated HCCs. The right atrial tumor was fixed to the atrial wall and arose from sites on the endocardium of the RA (Fig. 3A and B). The small tumor in the IVC was similarly fixed to the endothelium (Fig. 3C and D).

## Discussion

The mechanism of cardiac metastases is as a contiguous extension from the intrahepatic HCC via a tumor thrombus to the IVC or by lymphatic or hematologous spread. The majority of cardiac metastases are direct and contiguous extensions of the intrahepatic HCC. Isolated cardiac metastases that are discontinuous with an intrahepatic HCC are extremely rare. The literature was searched for descriptions of isolated cardiac metastases of HCC and 17 patients were identified (Table I) with a mean age of 58±13 years. In total, 15 patients (88.2%) had symptoms, with 13 (76.5%) suffering dyspnea. Intermittent obstruction by tumors in the cardiac cavity led to the symptoms of ball valve thrombus syndrome, which is able to induce sudden cardiac arrest (18). However, in the present study, the patient had no symptoms since the diameter of the tumor was 2 cm.

Extracorporal echocardiography was useful for detecting the atrial tumor in the present case. Kanematsu *et al*(19) described a tumor thrombus of HCC in the IVC detected by CT and MRI. Yoshitomi *et al*(20) and Van Camp *et al*(21) found transesophageal echocardiography useful. In theses studies, cardiac metastases were diagnosed by UCG and CT in 10 (58.8%) and 4 (23.5%) patients, respectively. Cardiac metastases were identified at autopsy in the early cases. The HCC was initially treated in 13 (76.5%) of the 17 patients [hepatectomy, n=9; transcatheter arterial embolization (TAE), n=3; chemotherapy, n=3] and then the cardiac metastases were identified.

In the present study, the tumors in the RA and IVC were not continuous growths from the intrahepatic HCC in the patient. The two tumors were individually isolated and attached to the endocardium or endothelium, respectively. This metastatic pathway was considered to be comprised of two mechanisms. The first was the seeding of a blood flow onto the endocardium or endothelium from the intrahepatic HCC and the second was cancer embolisms of the small vessels under the endocardium and endothelium via arterial systemic spread. However, in the published cases, the mechanism associated with the metastases could not be clarified. Cardiac metastases were notably located in the right ventricle (RV), RA and left ventricle (LV) in 10 (58.8%), 5 (29.4%) and 2 (11.8%) patients, respectively. A greater number of metastases had invaded the RV than the RA. These findings indicate that the main mechanism is embolism via arterial systemic spread as massive myocardial involvement was described in 4 patients.

Surgical (22) or non-surgical approaches, including TAE (23), transcatheter arterial infusion chemotherapy (TAI) (24) and radiotherapy (25), have been undertaken to treat IVC/RA metastases. In the selected published cases, eight (47.1%) out of 17 patients underwent surgery to relieve symptoms. In the present study, the patient underwent TAI only, but succumbed to HCC progression five months later. Overall, all the patients had an extremely poor prognosis regardless of the treatment strategy. Chang *et al*(26) reported that thalidomide is a useful angiogenesis inhibitor for IVC/RA metastases and new molecular target drugs, such as Sorafenib, have since emerged (27). However, further studies involving a larger cohort of patients with IVC/RA tumor thrombi are required.

## Figures and Tables

**Table I:** Patients with isolated HCC metastases to the heart.

Case	Ref.	Age (years), gender	Diagnosis stage	Symptoms	Pretreatment for cardiac metastases	Pathology	Location of initial HCC	Treatment for cardiac metastase	Prognosis
1	3	52M	Autopsy	Dyspnea, heart murmur	None	NS	RA, endocardium	Supportive care	Succumbed after 1 month
2	4	55M	Autopsy	Dyspnea, cyanosis	None	Ed II–III	RA, LA, endocardium	Supportive care	Succumbed after 1 month
3	5	67M	Autopsy	Fever, cough, chest discomfort	None	Ed III	LV, epicardium	Supportive care	Succumbed after 2 months
4	6	73M	Autopsy	Heart murmur	None	Ed I–II	RA, RV	TAE	Succumbed after 1 month
5	7	51M	CT	Dyspnea, palpitation	Hepatectomy	NS	RV, myocardium	Surgery	Succumbed
6	7	29M	UCG	Dyspnea	Hepatectomy	Ed II	LA	Chemotherapy	Succumbed
7	8	71M	Autopsy	Consciousness disorder	Chemotherapy	Ed II	RV, LV, myocardium	NS	Succumbed after 0.5 months
8	9	76M	UCG	Dyspnea	Hepatectomy, MCT, chemotherapy	Ed I–II	RV, myocardium	Surgery	Succumbed after 6 months
9	10	49F	UCG	Dyspnea, palpitation, heart murmur	Hepatectomy	Ed I–II	RV, myocardium	Surgery	Alive at 21 months
10	11	67F	UCG	Dyspnea, heart murmur	Hepatectomy	Ed II	RV	TAE	Alive at 3 months
11	12	43M	UCG, CT	Dyspnea, cough	Hepatectomy	Ed I–II	RV	Supportive care	Succumbed after 20 days
12	13	45M	UCG, CT	Dyspnea, dizziness	Hepatectomy	NS	RV	Surgery	Alive at 3 months
13	14	65F	CT	Dyspnea	Hepatectomy	NS	RV	Surgery	Succumbed after 3 months
14	15	45F	UCG	Dyspnea, syncope	Hepatectomy, TAE	Ed II	RV	Surgery	Succumbed after 4 months
15	16	63F	UGC	Dyspnea	TAE	Ed IV	RA	Surgery	NS
16	17	74F	UCG	Dyspnea, syncope	TAE	NS	RV	Surgery	Succumbed after 4 months
17	Present study	66F	UCG	None	Chemotherapy	Ed II	RA, endocardium	TAI	Succumbed after 5 months

UCG, ultrasound cardiography; MCT, microwave coagulation therapy; TAE, transcatheter arterial embolization; Ed, Edmondson; NS, not stated; RA, right atrium; RV, right ventricle; LA, left atrium; LV, left ventricle; TAI, transcatheter arterial infusion chemotherapy; HCC, hepatocellular carcinoma.

## Abbreviations:

CT: computed tomography;
HCC: hepatocellular carcinoma;
MRI: magnetic resonance imaging
